# Supplementary material for: Rationing nursing care and organizational factors in intensive care units
Source: PLoS One. 2024 Jul 25;19(7):e0306313. doi: 10.1371/journal.pone.0306313 (PMC11271898; doi:10.1371/journal.pone.0306313)
Supplement: S1 Appendix — (PDF) [file pone.0306313.s001.pdf]

Sekcja A. Kwestionariusz PRINCA

Prosimy o wskazanie, jak często podczas siedmiu ostatnich zmian/dyżurów nie mogłaś/eś ukończyć każdej z 31 opisanych czynności pielęgniarskich z powodu braku zasobów (personelu lub czasu). Powinnaś/eś ocenić częstotliwość występowania poszczególnej czynności jako „Nigdy”, „Rzadko”, „Czasami” lub „Często” w oparciu o swoje doświadczenie zawodowe, umieszczając krzyżyk „X” w odpowiednim polu. Jeśli żaden z przydzielonych pacjentów podczas tych siedmiu zmian nie wymagał opisanej czynności pielęgniarskiej, powinnaś/eś wybrać „Nie dotyczy”. Jeśli nie byłaś/eś w stanie osobiście wykonać zadania, ale udało Ci się poprosić kogoś innego, aby zrobił to za Ciebie (np. inna pielęgniarka lub personel pomocniczy) zadanie to powinno być uznane za kompletne w drodze oddelegowania innej osoby.

Jak często zdarzało się to w ciągu ostatnich 7 roboczych zmian		Nie dotyczy	Nigdy	Rzadko	Czasami	Często
1.	nie można było przeprowadzić rutynowej higieny u pacjentów (np. kąpieli, higieny jamy ustnej, opieki stomatologicznej) lub zapewnić wykonanie tego zadania poprzez oddelegowanie innej osoby?					
2.	nie można było przeprowadzić rutynowej pielęgnacji skóry u pacjentów lub zapewnić wykonanie tego zadania poprzez oddelegowanie innej osoby?					
3.	nie można było zmienić w odpowiednim czasie pościeli zabrudzonej krwią lub płynami ustrojowymi lub zapewnić wykonanie tego zadania poprzez oddelegowanie innej osoby?					
4.	nie można było asystować pacjentowi potrzebującemu chodzenia/uruchamiania poza łóżkiem lub zapewnić wykonanie tego zadania poprzez oddelegowanie innej osoby?					
5.	nie można było uruchomić pacjenta ani zmienić pozycji pacjenta z ograniczoną mobilnością lub zapewnić wykonanie tego zadania poprzez oddelegowanie innej osoby?					
6.	nie można zapewnić szybkiej pomocy po opróżnieniu jelit lub pęcherza (na przykład toaletę krocza) lub zapewnić wykonanie tego zadania poprzez oddelegowanie innej osoby?					
7.	nie można było odpowiednio pomóc pacjentom niezdolnym do samodzielnego jedzenia ani picia niezależnie od sposobu przyjmowania pokarmu lub zapewnić wykonanie tego zadania poprzez oddelegowanie innej osoby?					
8.	nie można było wdrożyć środków promujących komfort fizyczny (np. terminowe podawanie leków przeciwbólowych, dostosowanie temperatury, masaż pleców lub karku) lub zapewnić wykonanie tego zadania poprzez oddelegowanie innej osoby?					
9.	nie było możliwości podania leków (w tym terapii dożylnej) zgodnie z zaleceniami i zasadami bezpiecznej farmakoterapii?					
10.	nie było możliwości podawania żywienia dojelitowego lub pozajelitowego zgodnie z zaleceniami i zgodnie z bezpiecznymi praktykami?					
11.	nie było możliwości prowadzenia opieki nad ranami (w tym zmianę opatrunków), zgodnie z zaleceniami lekarza / standardami jednostki lub gdy według ciebie było to wskazane?					
12.	nie było możliwości zmiany miejsca dostępu dożylnego, rurki i / lub opatrunków w określonym czasie zgodnie z zaleceniami lekarza / standardami jednostki lub gdy według ciebie było to wskazane?					

13.	nie było możliwości odpowiedniego przestrzegania zalecanych wytycznych dotyczących bezpiecznego obchodzenia się z pacjentem (np. korzystania ze sprzętu wspomagającego oraz podnośników i / lub dodatkowego personelu)?					
14.	nie było możliwości odpowiedniego przestrzegania zalecanych wytycznych dotyczących kontroli infekcji (np. higiena rąk, technika aseptyczna, izolacja)?					
15.	nie było możliwości zapewnienia odpowiedniego czasu edukacji, który według Ciebie był wskazany dla pacjenta i / lub jego rodziny?					
16.	nie można było odpowiednio przygotowania pacjentów do leczenia, badań lub procedur?					
17.	nie można było zaoferować odpowiedniego poziomu wsparcia emocjonalnego lub psychologicznego pacjentowi lub rodzinie, które w danym momencie było potrzebne?					
18.	nie można było monitorować stanu fizjologicznego pacjenta (np. parametry życiowe, wartości laboratoryjne) zgodnie z zaleceniami lekarza /standardami jednostki lub gdy według ciebie było to konieczne?					
19.	nie można było monitorować emocji i zachowania pacjenta (np. przestrzeganie zażywania leków, nawyki żywieniowe, kontakty społeczne, nastrój) zgodnie z zaleceniami lekarza / standardami jednostki lub gdy według ciebie było to koniecznie?					
20.	nie można było monitorować fizycznego bezpieczeństwa pacjenta zgodnie z zaleceniami lekarza / standardami jednostki lub gdy według ciebie było to koniecznie?					
21.	nie można było śledzić zmian stanu pacjenta, niezrealizowanych próśb o interwencję u pacjenta (w tym oceny lub skierowania) lub niejasne zlecenia?					
22.	trzeba było trzymać w oczekiwaniu pacjenta lub członka rodziny dłużej niż 5 minut od momentu zasygnalizowania danej prośby (np. przez światło wywoławcze)?					
23.	nie można było odbyć ważnej rozmowy z innym członkiem wielodyscyplinarnego zespołu w sprawie dotyczącej opieki nad pacjentem, lub rozmowa ta została opóźniona?					
24.	nie można było odbyć ważnej rozmowy z zewnętrzną jednostką w sprawie dotyczącej opieki nad pacjentem, lub rozmowa ta została opóźniona?					
25.	nie można było odbyć ważnej rozmowy z pacjentem lub członkiem rodziny o potrzebach lub instrukcjach związanych z wypisem, czy też rozmowa została opóźniona?					
26.	nie było możliwości zapewnienia odpowiedniego nadzoru lub śledzenia wykonywania delegowanych działań przez inne osoby?					
27.	nie było możliwości odpowiedniego przejrzenia interdyscyplinarnej dokumentacji pacjenta, aby uzyskać złożone informacje na temat pacjenta?					
28.	nie było możliwości udokumentowania początkowego lub zmienionego planu opieki?					
29.	nie było możliwości udokumentowania wszystkich działań związanych z oceną i monitorowaniem stanu pacjenta?					
30.	nie było możliwości udokumentowania odpowiednio szczegółowo całego procesu wykonanej opieki pielęgniarskiej?					

31.	nie było możliwości właściwego ocenienia planu opieki (używając krytycznego myślenia), aby określić stosowność i / lub skuteczność interwencji i dokonać zmian jakie wskazano?					

Sekcja B. Ogólne doświadczenie zawodowe

1. Ocena pielęgniarska jakości opieki nad pacjentem
Zakreśl kołem liczbę, która wskazuje na standardową jakość opieki nad pacjentami w Twoim oddziale?

1 2 3 4 5 6 7 8 9 10

Niebezpiecznie niska jakość — Bezpieczna, ale niewiele więcej — Bardzo wysoka

2. Ogólna satysfakcja z pracy
Zakreśl kołem liczbę, która wskazuje na stopień zadowolenia ze swojej obecnej pracy pielęgniarskiej, biorąc pod uwagę wszystkie aspekty pracy, w tym także własne wartości, ideały i cele?

1 2 3 4 5 6 7 8 9 10

Jest tragiczna! — Jestem zadowolona — Uwielbiam to!
