# Supplementary material for: Rationing nursing care and organizational factors in intensive care units
Source: PLoS One. 2024 Jul 25;19(7):e0306313. doi: 10.1371/journal.pone.0306313 (PMC11271898; doi:10.1371/journal.pone.0306313)
Supplement: S1 Table — (DOCX) [file pone.0306313.s003.docx]

Tabel. Distribution of responses in hospitals.
