# Supplementary material for: Rationing nursing care and organizational factors in intensive care units
Source: PLoS One. 2024 Jul 25;19(7):e0306313. doi: 10.1371/journal.pone.0306313 (PMC11271898; doi:10.1371/journal.pone.0306313)
Supplement: Attachment — Submitted filename: Response to Reviewers.docx [file pone.0306313.s004.docx]

| Review Comments to the Author | Response |
| --- | --- |
| **Reviewer 1.** |  |
| The phrase " the organizational dimensions" could be removed from the title. Additionally, it is better to add the phrase "and organizational factors" | We changed the title to: Rationing nursing care and organizational factors in intensive care units |
| Please add the study time | We added the study time in the abstract, It is also present in study design |
| line 30: If the journal guidelines allow, remove the word "discussion" | We removed the word discussion |
| in general, I did not understand why the authors addressed only the number of beds and the type of hospital among the organizational factors. Because I think other organizational factors can contribute to the rationing of nursing care. The authors have also collected data on the number of work shifts, working hours, and patients under care, which are also considered organizational factors. Therefore, I wonder why the organizational factors were limited only to the number of beds and the type of hospital. I suggest that if there is no specific logic for choosing these two types of factors, it is better to state the organizational factors instead of emphasizing these two factors. In addition, it is better to clearly define the organizational factors in the introduction section. | We changed it in the Aims to: This study aimed to identify the extent of rationing of nursing care in intensive care units in different types of hospitals and determine the relationship between rationing of nursing care and hospital and staff characteristics. |
| The introduction section is not written logically and coherently. It does not show the importance and necessity of conducting the study | We improved the introduction, it’s completely reworded |
| A brief review of the literature on the research topic is necessary. | We added citation |
| I think that nursing workload in ICUs is a key factor in rationing nursing care. Therefore, I suggest that the introduction be developed using a related literature review of nursing workload in ICUs. For example, authors can use the following articles: <https://onlinelibrary.wiley.com/doi/full/10.1002/nop2.785> <https://onlinelibrary.wiley.com/doi/abs/10.1111/nicc.12548> | We added this factor and citations. |
| Method section: I was surprised to see so many decimals in this manuscript. Please enter numbers to two decimal places in all sections of the manuscript. | We changed it as suggested |
| Please explain how to estimate the sample size. | Sample size was determined using arithmetic mean estimation of PRINCE questionnaire |
| Line 80: Is the number 14 above 2014 related to the reference? Please correct it. | We removed the numer 14 |
| I strongly recommend using appropriate regression models in data analysis. | For this purpose, a stepwise multiple linear regression analysis was performed in both types of hospitals. Determination coefficients were also calculated using the formula: R^2^ = r^2^x100% (R² - coefficient of determination; r – Pearson’s correlation coefficient). |
| I emphasize the data analysis must be checked by a statistical consultant. | The data analysis was checked by a statistical consultant |
| Table 1: please merge the (N) and (%) columns. as follows: N (%). | we connected the columns |
| Table 1: please delete the percent (%) from the numbers. for example, write 88.78 instead of 88.78%. | We deleted the percent % |
| Table 2: Does the letter M mean "Mean"? Please write in full. | We changed it as suggested |
| Table 2: Please merge "M" and "SD" columns. | we connected the columns |
| Table 2: Please write the p-value of 0.00000000005 as below. P <001. AS WELL AS, please apply this comment for table 5. This type of P-value reporting is not correct. In such a situation, the follow-up is reported as P <001. Please apply this comment in similar sections as well. | We changed it as suggested |
| I suggest that the discussion section be developed using a related literature review. For example, authors can use the following articles: <https://onlinelibrary.wiley.com/doi/full/10.1002/nop2.785> <https://onlinelibrary.wiley.com/doi/abs/10.1111/nicc.12548> | We added that in discussion section. |
| **Reviewer 2.** |  |
| The study is conducted in a specific region of Poland, which may limit the applicability of its findings to other regions or countries with different healthcare systems and organizational structures. Future research could benefit from a broader geographical scope to enhance the generalizability of the results. Maybe add this point to the limiatation section. | We added it to limitations section:  Expanding the geographical range of subsequent studies could improve the applicability of the findings across diverse regions |
| While the quantitative approach provides a solid overview of the extent of care rationing, integrating qualitative methods could offer deeper insights into the reasons behind care rationing and the subjective experiences of nursing staff. This could include interviews or focus groups with nurses to explore the nuances of organizational factors affecting care rationing.  The cross-sectional design of the study provides a snapshot of the situation but does not account for changes over time. A longitudinal study design could offer insights into how organizational changes within hospitals affect the rationing of nursing care over time. | We added this to limitations section:  Future research could benefit from incorporating qualitative methods, such as interviews or focus groups, to gain deeper insights into the organizational factors influencing care rationing. Additionally, the study's cross-sectional design provides only a temporal glimpse, limiting the ability to observe how the rationing of nursing care evolves over time. A longitudinal study design could be instrumental in understanding the impact of organizational changes on care rationing within hospitals over extended periods. |
| The manuscript states that all relevant data are within the manuscript and its Supporting Information files. Ensuring the accessibility of this data for verification and future research would enhance the manuscript's transparency and credibility. | All relevant data is available in supporting files |
