# Supplementary material for: Rationing nursing care and organizational factors in intensive care units
Source: PLoS One. 2024 Jul 25;19(7):e0306313. doi: 10.1371/journal.pone.0306313 (PMC11271898; doi:10.1371/journal.pone.0306313)
Supplement: Attachment — Submitted filename: Response to Reviewer.docx [file pone.0306313.s005.docx]

| Page 2, line 19: please change “Omitted nursing care” to “rationing of nursing care” | We changed it |
| --- | --- |
| Page 2, lines 21-22: Please add the abbreviation “intensive care units” in parentheses as follows: Intensive Care Units (ICUs). | We changed it |
| Page 2, line 40: please use the “ICUs” instead of the “intensive care units”. | We changed it |
| Page 2, line 41: add the term of “organizational factors” to keywords. | We changed it |
| Page 3, lines 44-45: I suggest removing the abbreviations MCN and MNCM because they are used only once in the entire manuscript. | We changed it |
| • Page 7, line 147: Please write "neonatal ICUs" instead of "ICUs for neonates". | We changed it |
| • I suggest that the text of lines 156 to 162 be moved to the ethical considerations section and merged with it so that sentences do not become repetitive. Because some sentences overlap. | We moved it to ethical section |
| Table 1: Please mention the age unit in the related column in the table in parentheses as follows: age (years) | We changed it |
| Table 1, the column related to "the number of hours spent at work per month": please put the word "hours" in parentheses in the first column and remove it from the rest of the columns as follows: number of hours spent at work per month (hours): write "100-180" instead of "100-180 hours" | We changed it |
| Table 3: Please mention the age unit in the related column in the table in parentheses as follows: age (years) | We changed it |
|  |  |
|  |  |
